# Cost-effectiveness of a Telemonitoring Program for Patients With Heart Failure During the COVID-19 Pandemic in Hong Kong: Model Development and Data Analysis

**DOI:** 10.2196/26516

**Published:** 2021-03-03

**Authors:** Xinchan Jiang, Jiaqi Yao, Joyce Hoi-Sze You

**Affiliations:** 1 School of Pharmacy Faculty of Medicine The Chinese University of Hong Kong Hong Kong China (Hong Kong)

**Keywords:** telemonitoring, mobile health, smartphone, heart failure, COVID-19, health care avoidance, cost-effectiveness

## Abstract

**Background:**

The COVID-19 pandemic has caused patients to avoid seeking medical care. Provision of telemonitoring programs in addition to usual care has demonstrated improved effectiveness in managing patients with heart failure (HF).

**Objective:**

We aimed to examine the potential clinical and health economic outcomes of a telemonitoring program for management of patients with HF during the COVID-19 pandemic from the perspective of health care providers in Hong Kong.

**Methods:**

A Markov model was designed to compare the outcomes of a care under COVID-19 (CUC) group and a telemonitoring plus CUC group (telemonitoring group) in a hypothetical cohort of older patients with HF in Hong Kong. The model outcome measures were direct medical cost, quality-adjusted life-years (QALYs), and incremental cost-effectiveness ratio. Sensitivity analyses were performed to examine the model assumptions and the robustness of the base-case results.

**Results:**

In the base-case analysis, the telemonitoring group showed a higher QALY gain (1.9007) at a higher cost (US $15,888) compared to the CUC group (1.8345 QALYs at US $15,603). Adopting US $48,937/QALY (1 × the gross domestic product per capita of Hong Kong) as the willingness-to-pay threshold, telemonitoring was accepted as a highly cost-effective strategy, with an incremental cost-effective ratio of US $4292/QALY. No threshold value was identified in the deterministic sensitivity analysis. In the probabilistic sensitivity analysis, telemonitoring was accepted as cost-effective in 99.22% of 10,000 Monte Carlo simulations.

**Conclusions:**

Compared to the current outpatient care alone under the COVID-19 pandemic, the addition of telemonitoring-mediated management to the current care for patients with HF appears to be a highly cost-effective strategy from the perspective of health care providers in Hong Kong.

## Introduction

Heart failure (HF) is a chronic disease affecting 38 million patients worldwide, with high in-hospital mortality (6.4%), 1-year readmission rate (24%-30%), and 1-year postdischarge mortality (20%) [1-5]. This chronic cardiac disease imposes a substantial global economic burden of US $108 billion per annum (approximated in 2012) [6], which is expected to increase considerably with the aging of the population [7]. Hong Kong is a developed city with an aging population, and the local epidemiological findings on outcomes of patients with HF were consistent with those of western countries [8,9].

The COVID-19 pandemic has imposed major burdens and barriers on the operation of health care systems worldwide. COVID-19 has not only disrupted the provision of routine medical care but has also caused patients to delay and avoid seeking medical care [10]. COVID-19 was reported to be a factor associated with avoiding medical consultation in Hong Kong [11]. Patients with chronic conditions such as HF are therefore at risk of suboptimal care during the COVID-19 pandemic as a result of disruption or avoidance of routine medical care. The treatment outcomes of HF under current care during the COVID-19 pandemic are expected to be compromised.

Telehealth is a potential timely alternative to minimize the risk of COVID-19 transmission by reducing direct physical contact and to sustain continuous medical care to patients with HF during the COVID-19 pandemic [12]. The benefits of telemonitoring programs have been examined in clinical studies for the management of patients with HF. A meta-analysis reported that the application of telemonitoring program was associated with reduced risk of all-cause mortality and HF-related mortality [13].

The Markov model is a well-established decision-analytic model for simulation of expected treatment costs and health-related outcomes by incorporating relevant clinical probabilities, costs, and utility inputs. In a Markov model, hypothetical subjects proceed through health states (Markov states) in the next model cycle according to transition probabilities. Markov modeling is recommended for evaluating the outcomes of diseases that might progress, improve, or relapse through transition over a series of health states [14]. The cost-effective application of telemonitoring for the management of HF was demonstrated by Markov model–based analyses prior to the era of COVID-19 [15,16], and the patients’ medical avoidance was therefore not evaluated as an influential factor. In this study, COVID-related medical avoidance was considered in the model-based analysis. The aim of our study was to examine the potential clinical and health economic outcomes of adding telemonitoring programs to current medical care during the COVID-19 pandemic for the management of patients with HF from the perspective of health care providers in Hong Kong.

## Methods

### Model Design

**Figure 1:**
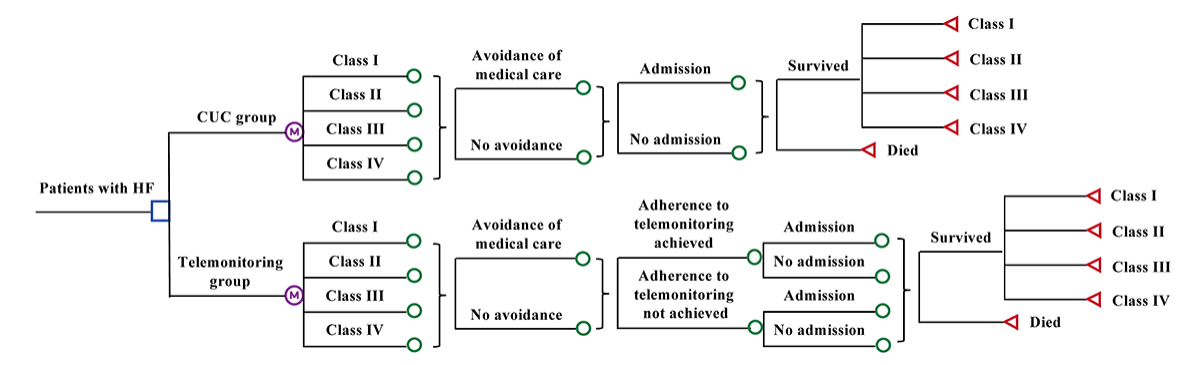
Simplified Markov model of telemonitoring for patients with HF. CUC: care under COVID-19; HF: heart failure.

A Markov decision-analytic model was designed to estimate the potential outcomes of current care under COVID-19 (CUC) with and without telemonitoring in a hypothetical cohort of older patients with HF (age 65 years or above) in Hong Kong (Figure 1). The outcomes were simulated from the entry of the model for a time frame of 10 years or until death, whichever occurred first. The two strategies examined in this study were (1) CUC plus telemonitoring (telemonitoring group) and (2) CUC alone (CUC group). The hypothetical cohort entered the model at one of the New York Heart Association (NYHA) classes I-IV and proceeded to another health status by the corresponding probability in each monthly cycle. The model outcome measures were direct medical cost, quality-adjusted life-years (QALYs), and incremental cost-effectiveness ratio (ICER).

Multidisciplinary care is the standard management approach in usual care for patients with HF in Hong Kong, as recommended by the American College of Cardiology Foundation/American Health Association Guideline for the Management of Heart Failure [17]. Patients in the CUC and telemonitoring groups therefore all received multidisciplinary care, while patients in telemonitoring group received telemonitoring-mediated HF management in addition to multidisciplinary care. The telemonitoring-mediated management approach evaluated in a clinical outcome study was adopted in this model [18]. The patients in the telemonitoring group transmitted cardiac measures (heart rate, blood pressure, and weight) daily to the HF management team and answered a short series of questions pertinent to their HF symptoms via an app downloaded to a smartphone. A clinically validated algorithm that was embedded in the app stratified patients into different states and further identified patients with urgent needs. The patients with urgent needs would receive an alert message and an automated call suggesting emergent services. The on-call clinician would also be alerted to provide timely intervention at the onset of symptom exacerbations. Patients who were classified as nonurgent cases would receive self-instruction on administration of medications and when to contact a care provider.

Because of patients’ concerns about the risk of acquiring COVID-19 at health care facilities during the pandemic, patients in both arms might or might not have avoided attending the in-person medical care clinic. The telemonitoring-mediated care also required daily transmission of cardiac measures via a smartphone app, and patients in the telemonitoring group might or might not have achieved adherence to the telemonitoring requirements. Patients in both arms might have experienced HF-related hospitalization. For the patients who survived (with or without hospitalization) in each cycle, they might have remained in the same NYHA classification or improved/progressed to another NYHA classification.

### Model Inputs

All the model inputs are shown in Table 1. The clinical inputs were retrieved from published reports written in English, identified from a literature search on MEDLINE over the period of 2000-2020. Epidemiology or disease burden studies in the Chinese population, randomized clinical trials, and meta-analyses were the preferred sources for clinical model inputs.

**Table 1:** Model parameters.

Parameters			Base case value	Range of sensitivity analysis	Distribution		Reference	
**Clinical inputs**								
	**Proportion of NYHA^a^ classification (%)**					Dirichlet		[19]
		Class I	9	8.1-9.9				
		Class II	44	39.6-48.4				
		Class III	34	30.6-37.4				
		Class IV	13	8.6-17.4				
	**Transition probability (monthly)**					Dirichlet		[20]
		I to I	0.9597	0.9538-0.9678				
		I to II	0.0394	0.0315-0.0473				
		I to III	0.0009	0.0007-0.0011				
		I to IV	0	0-0.0011				
		II to I	0.0073	0.0058-0.0088				
		II to II	0.9877	0.9852-0.9902				
		II to III	0.0039	0.0031-0.0047				
		II to IV	0.0011	0.0009-0.0013				
		III to I	0.001	0.0008-0.0012				
		III to II	0.0443	0.0354-0.0532				
		III to III	0.8843	0.8612-0.9074				
		III to IV	0.0704	0.0563-0.0845				
		VI to I	0.0010	0.0008-0.0012				
		VI to II	0.0443	0.0354-0.0532				
		VI to III	0.8515	0.8612-0.9074				
		VI to IV	0.1032	0.0563-0.0845				
	Probability of HF^b^-related hospitalization in multidisciplinary care (monthly)		0.0296	0.0237-0.15	Beta		[9]	
	Probability of all-cause mortality in multidisciplinary care (monthly)		0.0279	0.0076-0.0383	Beta		[9]	
	**Risk ratio of event with** **versus** **without multidisciplinary care**							
		HF-related hospitalization	0.74	0.64-0.87	Lognormal		[21]	
		All-cause mortality	0.75	0.59-0.96	Lognormal		[21]	
	**Risk ratio of event with versus without telemonitoring**							
		HF-related hospitalization	0.5	0.36-0.64	Lognormal		[18]	
		All-cause mortality	0.81	0.70-0.94	Lognormal		[13]	
	Adherence to telemonitoring-guided management (%)		80	64-96	Triangular		[22]	
	COVID 19–related health care avoidance (%)		26.1	21-31.5	Triangular		[11]	
	Duration of COVID 19–related health care avoidance (years)		1.5	0.5-2	Triangular		[23]	
**Utility inputs**								
	**Utilities**					Uniform		[24]
		NYHA class I	0.82	0.78-0.85				
		NYHA class II	0.74	0.69-0.75				
		NYHA class III	0.64	0.55-0.77				
		NYHA class IV	0.46	0.41-0.61				
	**Disutilities of hospitalization**					Uniform		[24]
		NYHA class I	0.04	0.03-0.05				
		NYHA class II	0.07	0.06-0.08				
		NYHA class III	0.10	0.08-0.12				
		NYHA class IV	0.29	0.23-0.35				
**Cost** **inputs**								
	Daily cost of hospitalization (US $)		654	523-785	Gamma		[25]	
	Length of hospitalization for HF (days)		8	6-10	Triangular		[26]	
	Monthly outpatient cost for HF (US $)		197	158-236	Gamma		[27]	
	**Telemonitoring-mediated care (US $)**							
		Site implementation cost per patient	80	64-96	Gamma		[16]	
		Monthly cost of telemonitoring	50	40-60	Gamma		[16]	

^a^NYHA: New York Heart Association.

^b^HF: heart failure.

At the entry of the model, the distribution of patients among the four statuses (NYHA class I: 9%, NYHA class II: 44%, NYHA class III: 34%, and NYHA class IV: 13%) adopted the baseline characteristics of patients with HF in Northeast Asia [19]. The yearly transition rates between NYHA classes were retrieved from the Eplerenone in Mild Patients Hospitalization And Survival Study in Heart Failure [20], and MATLAB (MathWorks) was used to generate the monthly transition matrix. HF-related hospitalization (2.96%) and all-cause mortality for patients aged ≥65 years (2.79%) with multidisciplinary care were approximated from the Hong Kong Heart Failure Registry. In this study, a total of 1940 new-onset HF cases were identified in the Hong Kong Chinese population between 2005 and 2012. Both of the above estimates were retrieved from patients followed in the outpatient setting, with a prior history of hospitalization for decompensated HF [9]. The clinical impacts of multidisciplinary care (vs without multidisciplinary care) on HF-related admission (risk ratio [RR] 0.74; 95% CI 0.63-0.87) and all-cause mortality (RR 0.75; 95% CI 0.59-0.96) were retrieved from a systematic review of 29 trials (5039 patients) on multidisciplinary strategies for management of patients with HF [21]. The probabilities of HF-related hospitalization and all-cause mortality in patients who avoided medical care during the COVID-19 pandemic were approximated using the risks of events without multidisciplinary care. The relative change of hospitalization rate associated with telemonitoring-medicated care (RR 0.5, 95% CI 0.36-0.64) was obtained from an outcome study of a smartphone-based telemonitoring system in 315 patients with HF [18]. The relative impact of telemonitoring on all-cause mortality (RR 0.81, 95% CI 0.70-0.94) was estimated from a meta-analysis of 37 trials that evaluated the comparative effectiveness of telemonitoring versus no telemonitoring for HF management [13]. The adherence of telemonitoring was defined as achieving 70% of scheduled daily data transmission and HF symptom reporting. The percentage of achieved adherence was assumed to be 80% based on a study investigating the patient adherence of a smartphone-based telemonitoring system for HF [22]. The percentage of medical avoidance among patients with HF (26.1%) was approximated from a public survey of 765 subjects on the use of health services during the COVID-19 pandemic in Hong Kong [11]. The base-case value of health care avoidance duration was estimated to be 1.5 years with a range of 0.5-2 years, based upon the epidemiologic projections of the COVID-19 pandemic [23].

Both the utility scores of the NYHA classes and disutilities due to hospitalization were retrieved from the predicted utilities of patients with HF in the Systolic Heart Failure Treatment with the I*_f_* Inhibitor Ivabradine Trial (n=5313) [24]. The expected QALY gain in each group was calculated by the time spent in the health statuses and the corresponding utility scores. The QALY gain was discounted at an annual rate of 3%.

The cost analysis in this model was conducted using direct medical costs in the year 2020 from the perspective of public health care providers in Hong Kong. The costs of telemonitoring-medicated care (in the telemonitoring group) and the costs of HF-related inpatient and outpatient care (in both groups) were included. The cost of HF-related hospitalization was estimated by the daily cost of inpatient care and the length of stay of the patients. The daily cost of inpatient care was approximated from the fees and charges of public hospital services provided by the Hospital Authority in Hong Kong [25]. The length of hospital stay was estimated from a review on the burden of HF in 9 countries or regions (including Hong Kong) in Asia [26]. The monthly outpatient cost was estimated from the findings of a retrospective observational study on the total management cost (including hospitalization cost and ambulatory care cost) of patients with HF (n=73) recruited from a public hospital in Hong Kong [27]. The implementation cost of telemonitoring per capita (US $80) and monthly cost of telemonitoring (US $50) were approximated from the reported costs of a smartphone-based telemonitoring system [16], including a smartphone, blood pressure monitor, weight scale, and licensing fee. The implementation cost was a one-time charge, while the monthly cost of telemonitoring was a recurrent cost for maintenance of the app. Hong Kong is a developed city with a high smartphone penetration rate of 85.5% in the overall population [28]. In this study, the monthly cost of telemonitoring was estimated at US $50 (US $1=HK $7.8), assuming the patients used their smartphones and installed the telemonitoring app. All costs were discounted annually by 3%.

### Cost-effectiveness Analysis and Sensitivity Analysis

Expected costs and QALY gains were simulated for the two strategies in the base-case analysis. The ICERs were calculated using the equation (total cost_telemonitoring group_–total cost_CUC group_)/(QALY_telemonitoring group_–QALY_CUC group_). As recommended by the World Health Organization in 2002, an ICER less than 1 × the gross domestic product per capita was considered to be highly cost‐effective [29]. The gross domestic product per capita of Hong Kong was US $48,937 in 2019 and was adopted as the willingness‐to‐pay (WTP) threshold [30]. A treatment alternative was preferred if (1) it was effective in saving QALYs at lower cost or (2) it was effective in saving QALYs at a higher cost with an acceptable ICER (< the WTP threshold).

Deterministic and probabilistic sensitivity analyses using Monte Carlo simulations were performed to examine the robustness of the base-case results. In the deterministic sensitivity analysis, each model input was evaluated over the range reported in the retrieved studies. If no range was specified, the parameter was examined over a range of ±20% of the base-case value. In the probabilistic analysis, 10,000 Monte Carlo simulations of each model outcome measure were generated by randomly drawing the value of all model inputs simultaneously from the distribution specified in Table 1. The probabilities of each strategy to be accepted as cost-effective in the 10,000 Monte Carlo simulations were determined against the variation of the WTP threshold (from US $0-100,000/QALY) in the acceptability curve. All analyses were performed using TreeAge Pro 2020 (TreeAge Software, Inc).

## Results

### Changes of Outcomes With Versus Without COVID-19–Related Health Care Avoidance

Over a time frame of 1.5 years (base-case value of health care avoidance duration), the expected direct medical cost and QALYs of the CUC group (with COVID-19–related health care avoidance) were US $7114 and 0.7960 QALYs, respectively. The expected cost and QALYs of usual care (without COVID-19–related health care avoidance) over a period of 1.5 years were US $6888 and 0.8135 QALYs, correspondingly. Compared with usual care (without COVID-19–related health care avoidance), CUC (with COVID-19–related health care avoidance) increased the cost by US $226 with a loss of 0.0175 QALYs.

### Base-Case Analysis

The expected QALY gains and total costs of the telemonitoring group and the CUC group were compared. The direct medical cost for the CUC group was US $15,603 and the QALYs were 1.8345, while these values for the telemonitoring group were US $15,888 and 1.9007, respectively. The incremental QALYs saved by the telemonitoring group (versus the CUC group) were 0.0662, with an additional cost of US $284. The ICER for the telemonitoring group versus the CUC group was US $4292/QALY, which is below the WTP threshold of 48,937 USD/QALY (1× gross domestic product per capita in Hong Kong). Telemonitoring was therefore a highly cost-effective strategy in the base-case analysis.

### Sensitivity Analyses

**Figure 2:**
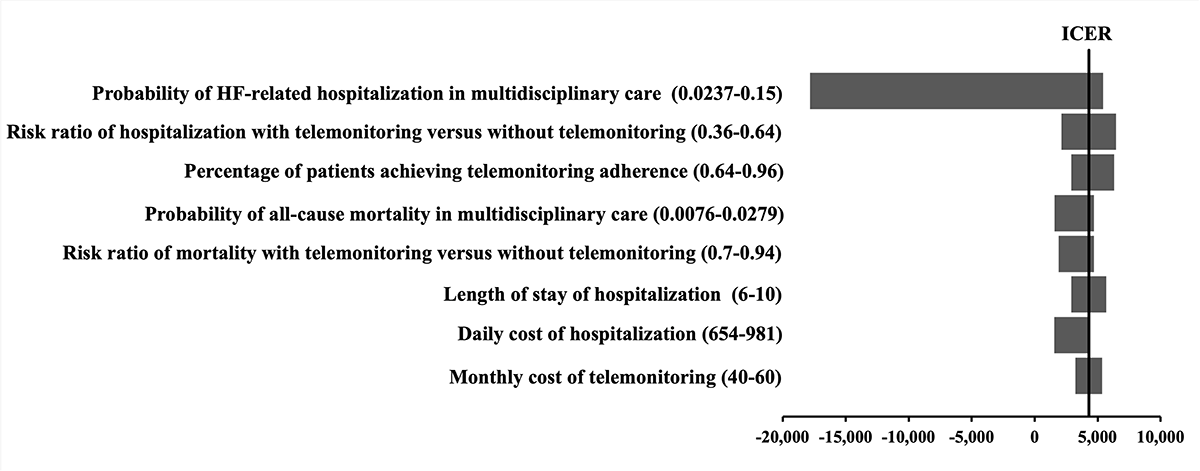
One-way sensitivity analysis of the ICER of the telemonitoring group versus the CUC group. CUC: care under COVID-19; ICER: incremental cost-effectiveness ratio.

One-way deterministic sensitivity analyses were conducted for all model inputs. The ICERs of the telemonitoring group remained below the WTP threshold in the one-way variation of all parameters. No influential factor with the threshold value was found. For eight critical parameters, the ICERs varied by more than 20% (Figure 2): probability of HF-related hospitalization in multidisciplinary care, risk ratio of hospitalization with telemonitoring versus without telemonitoring, percentage of patients achieving telemonitoring adherence, probability of all-cause mortality in multidisciplinary care, risk ratio of mortality with telemonitoring versus without telemonitoring, length of stay of hospitalization, daily cost of hospitalization, and monthly cost of telemonitoring. Of these eight critical parameters, the probability of HF-related hospitalization in multidisciplinary care had the highest impact on the total cost. When the monthly probability of HF-related hospitalization in multidisciplinary care increased from the base-case value of 0.0296 to >0.0515, the telemonitoring group gained higher QALYs at a lower cost than the CUC group.

**Figure 3:**
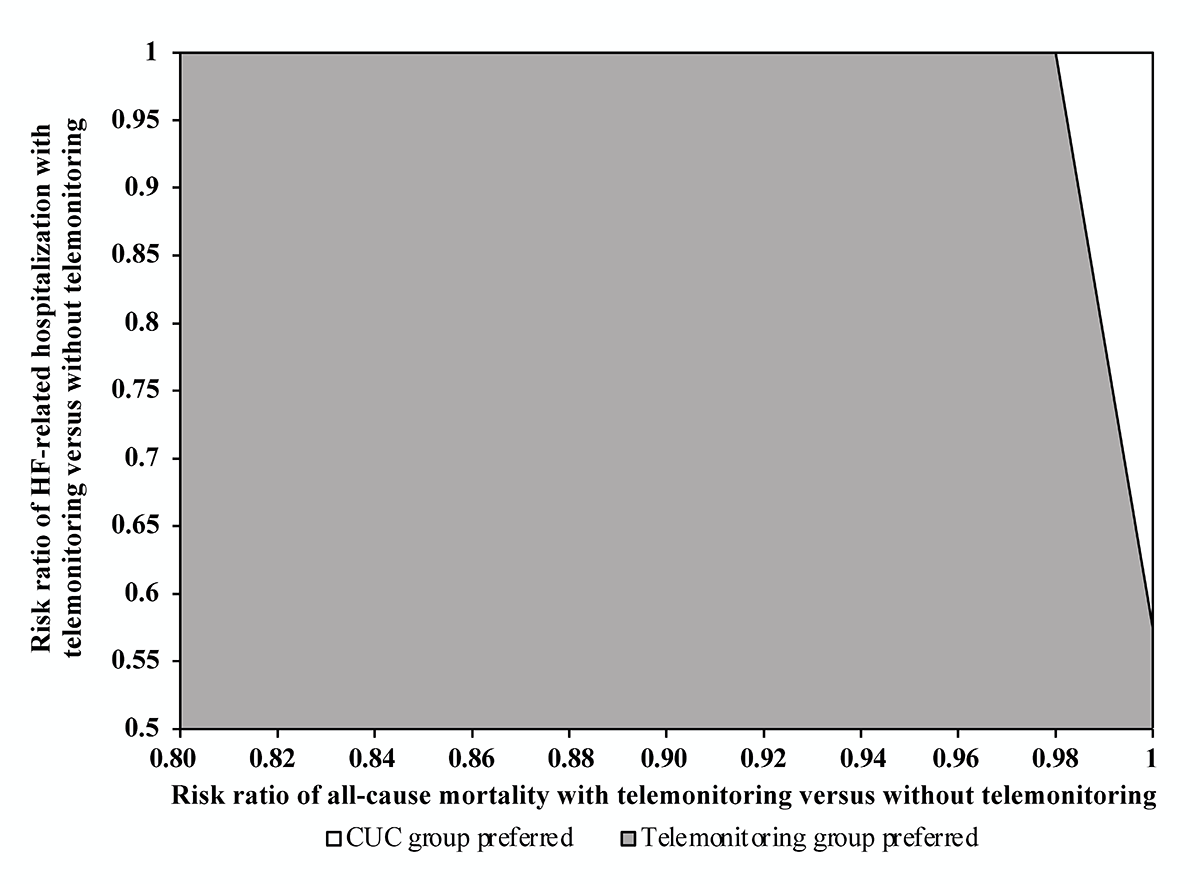
Two-way variation of the risk ratios with telemonitoring versus without telemonitoring on HF-related hospitalization and all-cause mortality. CUC: care under COVID-19; HF: heart failure.

The risk ratios of telemonitoring versus no telemonitoring for HF-related hospitalization and all-cause mortality were two parameters representing the relative effectiveness of the telemonitoring-mediated care. To further investigate the interaction of these two parameters with the cost-effective acceptance of telemonitoring, a two-way deterministic sensitivity analysis was conducted with the risk ratios of telemonitoring versus without telemonitoring for HF-related hospitalization (range 0.5-1) and all-cause mortality (range: 0.81-1). The gray area in Figure 3 indicates the combinations of these two variables for telemonitoring to be acceptable as the preferred option (higher QALY gained at lower cost or at higher cost with an ICER< the WTP threshold).

**Figure 4:**
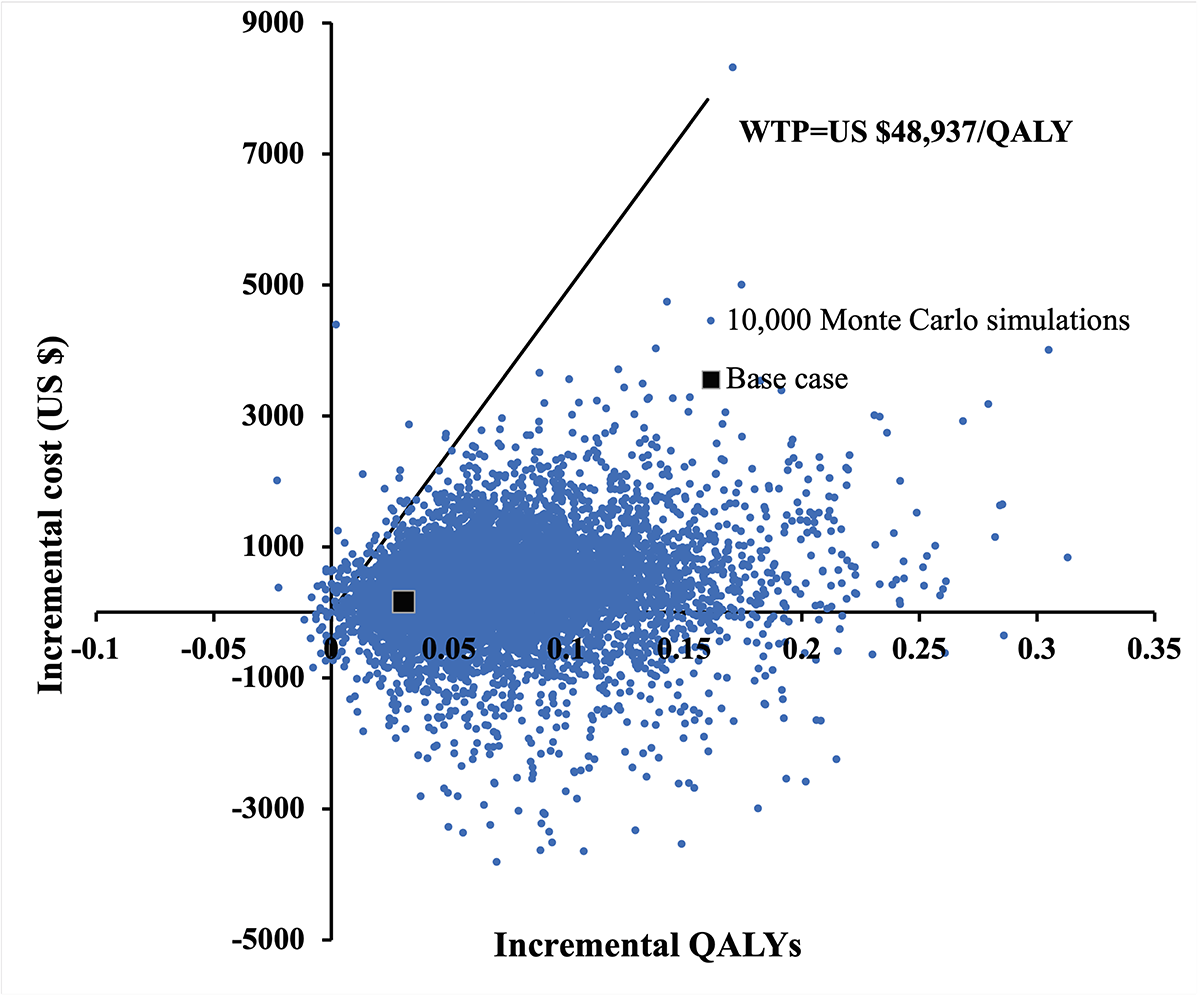
Scatter plot of the incremental cost-effectiveness ratios for the telemonitoring group versus the care under COVID-19 group. QALY: quality-adjusted life-year; WTP: willingness-to-pay.

The incremental costs versus incremental QALYs gained by telemonitoring (when compared with the CUC group) in 10,000 Monte Carlo simulations are shown in a scatter plot in Figure 4. The telemonitoring group gained an average QALY of 0.0688 (95% CI 0.0681-0.0695, *P*<.001), with a mean additional cost of US $319 (95% CI US $306-US $333, *P*<.001). In 10,000 Monte Carlo simulations, the probability of the telemonitoring group to be more effective in QALY gain and cost-saving was 23.5%. The telemonitoring group gained a higher QALY at a higher cost, with ICER<WTP (US $48,937/QALY) 75.7% of the time.

**Figure 5:**
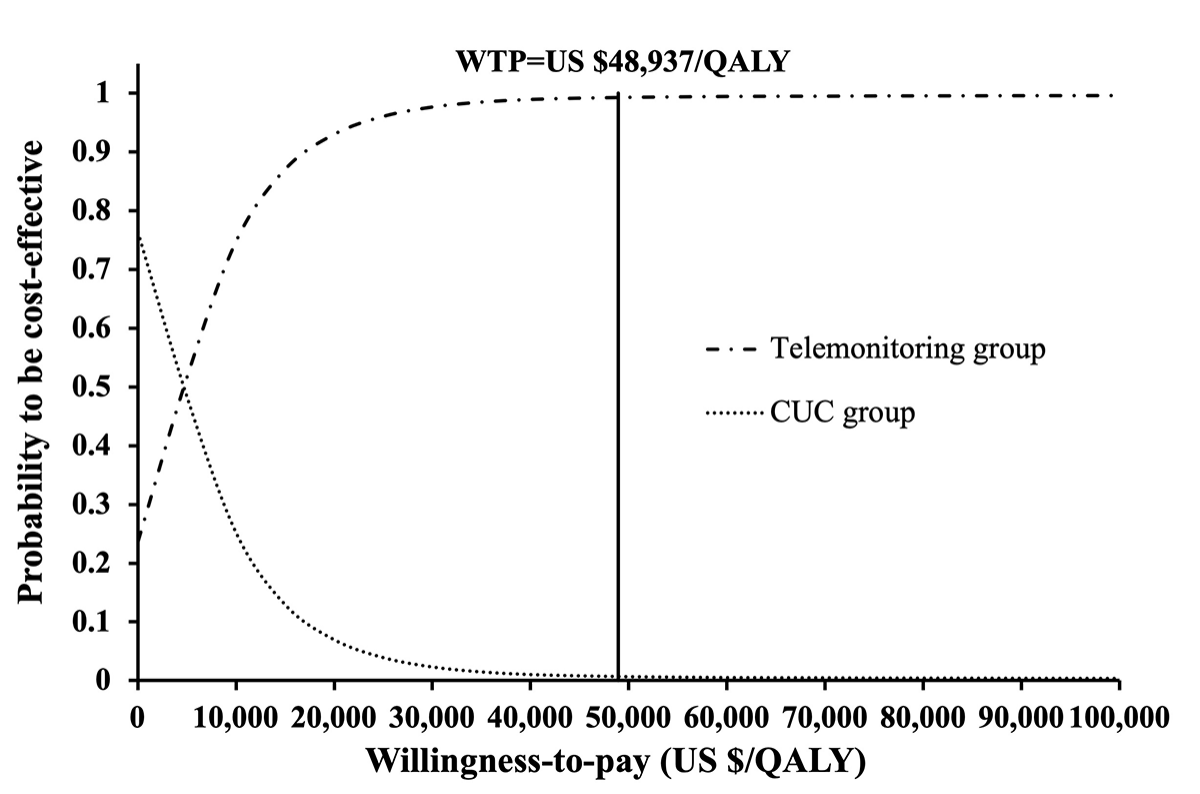
Cost-effectiveness acceptability curve for each strategy to be the preferred strategy against the WTP threshold. CUC: care under COVID-19; QALY: quality-adjusted life year; WTP: willingness-to-pay.

The probabilities of each strategy to be accepted as cost-effective are shown in the acceptability curve over a wide WTP range of US $0-100,000/QALY (Figure 5). The probabilities of the telemonitoring and CUC groups were the same (50%) at a WTP threshold of US $4700/QALY. The telemonitoring group was accepted to be cost-effective 99.2% of the time at the WTP threshold of US $48,937/QALY.

## Discussion

### Principal Results

This is the first analysis of the potential cost-effectiveness of smartphone-based telemonitoring systems for HF management during the COVID-19 pandemic. Our model results indicated that adding telemonitoring to current CUC for the management of patients with HF is a cost-effective strategy in the base-case analysis, with an ICER (US $4292/QALY) 10-fold below the WTP threshold (US $48,937/QALY). One-way sensitivity analysis supported the robustness of the base-case findings in that no influential parameter with a threshold value was identified. The high probability of the telemonitoring group to be accepted as the preferred strategy throughout a wide WTP range in the probabilistic sensitivity analysis further supported that adding telemonitoring to HF management is a highly cost-effective strategy.

The implementation cost is a modifiable factor when introducing a new technology in a health care system. In this study, telemonitoring was assumed to have a monthly cost of US $50 based on the estimated cost of a currently available smartphone-based telemonitoring system in Canada [16,18]. We further examined the impact of the monthly cost of the telemonitoring system in an extended one-way sensitivity analysis, and we found that telemonitoring-mediated care remained highly cost-effective if the monthly cost of telemonitoring was below US $467. Our findings were consistent with a cost-utility study of a telemonitoring-mediated HF care system in Canada in that the telemonitoring strategy was highly acceptable to be cost-effective, with an ICER of US $6701/QALY (WTP threshold=US $37,718/QALY) [16]. Our study further evaluated the interacting impact of two key parameters (risk ratios of events with telemonitoring vs without telemonitoring), which represented the relative effectiveness of telemonitoring in lowering HF-related hospitalization and all-cause mortality, on the cost-effective acceptance of the telemonitoring strategy. The combinations of these two parameters, as indicated in the two-way sensitivity analysis (Figure 3), provided the effectiveness thresholds required for the telemonitoring program to be accepted as cost-effective.

Health care systems in many countries worldwide are facing unprecedented challenges to maintaining routine medical care. This is particularly difficult when the target patients are older people with chronic cardiac diseases, who also belong to the high-risk group for life-threatening complications if they acquire COVID-19. In Hong Kong, the public health care system has struggled to provide care to patients with COVID-19 and protection against the disease to staff and other patients. Under these circumstances, public health care providers deferred some nonurgent care, and older patients also avoided attending their scheduled routine care appointments. As a result of fewer in-person clinic follow-ups, the risks of unplanned HF-related hospitalization and subsequently mortality inevitably increased.

The benefits of providing telemonitoring programs for HF management were recognized long before the COVID-19 pandemic. The pandemic has highlighted the urgency of adding telemonitoring-mediated care to in-person routine care for patients with HF [31]. Hong Kong is a developed city with a high smartphone penetration rate [28]. An effective smartphone-based telemonitoring system with a clinician-approved algorithm is a feasible and practical option for patients with HF in Hong Kong. In light of social distancing measures in the landscape of the COVID-19 pandemic, the acceptance of applying telemonitoring-mediated care is expected to highly increase at the levels of policy decision-makers, health care providers, and patients. The COVID-19 pandemic will surely catalyze the application of telemonitoring-mediated health care services in the very near future. Cost-effectiveness evaluation of telemonitoring-based medical care is therefore highly warranted to assist policy makers in the decision-making process of resource allocation.

### Limitations

There are limitations to this analysis. The cohort-based Markov model simplified real-life HF events with a limited number of health states. Other factors can impact the cost-effectiveness of HF management. For instance, influenza infection is associated with increased morbidity and mortality of patients with HF [32], and the influenza infection rate has dramatically decreased since the COVID-19 outbreak in Hong Kong [33]. Further evaluation of the impact of reduced influenza infections on HF outcome measures is highly warranted. The impact of telemonitoring on HF hospitalization and all-cause mortality varied among different types of telemonitoring, as indicated by the findings of a comprehensive network meta-analysis [13]. The cost-effectiveness of telemonitoring may therefore vary subject to the specific type of telemonitoring. Some model inputs were retrieved from overseas trials, which may affect the applicability of the model results for patients with HF in Hong Kong. Vigorous sensitivity analysis was therefore conducted on all model inputs over a broad range. The base-case results were found to be robust over the variation of all model inputs in both the deterministic and probabilistic sensitivity analyses. Additionally, the adherence of telemonitoring is not a parameter ready to be transferred between different health care systems. Health care practitioners should therefore examine the adherence of local patients when implementing a telemonitoring program for patients with HF.

### Conclusion

Compared to the current CUC during the pandemic alone, the addition of telemonitoring-mediated management to current care for patients with HF appears to be a highly cost-effective strategy from the perspective of health care providers in Hong Kong. Our findings provide evidence to inform decision makers on the application of telemonitoring amid the COVID-19 pandemic. Telemonitoring has long been considered as a future model of care, and the COVID-19 pandemic has fast-forwarded the application timeline of telemonitoring in clinical settings worldwide. It is expected that a mixed mode of disease management with in-person and telemonitoring-mediated care is likely to be sustained beyond the pandemic era. Further cost-effectiveness evaluations of mixed modes of care for the management of high-burden chronic diseases, such as diabetes mellitus, are highly warranted.

## Abbreviations

CUC: care under COVID-19
HF: heart failure
ICER: incremental cost-effective ratio
NYHA: New York Heart Association
QALY: quality-adjusted life-year
RR: risk ratio
WTP: willingness-to-pay
